# Characteristics of tertiary lymphoid structures in prostate cancer and the impact of neoadjuvant therapy on their formation and maturation

**DOI:** 10.3389/fimmu.2025.1663396

**Published:** 2025-11-04

**Authors:** Shengzhuo Liu, Yunfei Yu, Jing Zhou, Lucheng Yang, Xin Yan, Xiaoyang Liu, Kai Ma, Liangren Liu, Xianding Wang, Chengjian Zhao, Qiang Dong

**Affiliations:** ^1^ Department of Urology, Institute of Urology, West China Hospital, Sichuan University, Chengdu, Sichuan, China; ^2^ State Key Laboratory of Biotherapy and Cancer Center, West China Hospital, Sichuan University, and Collaborative Innovation Center for Biotherapy, Chengdu, Sichuan, China

**Keywords:** prostate cancer, organoid, tertiary lymphatic structures (TLSs), neoadjuvant hormonal therapy (NHT), immunotherapy

## Abstract

**Synopsis:**

Tertiary lymphoid structures (TLSs) are linked to better outcomes in prostate cancer. Neoadjuvant hormone therapy enhances TLS formation, immune cell infiltration, and tumor response—supporting the potential of combining hormone and immunotherapy for improved treatment.

**Purpose:**

This study investigates the characteristics and prognostic significance of tertiary lymphoid structures (TLS) in prostate cancer (PCa), and explores the impact of neoadjuvant hormone therapy (NHT) on TLS formation and maturation.

**Materials and methods:**

TLS features and immune infiltration were assessed in PCa cohorts using H&E and multiplex immunohistochemistry (mIHC) for Ki67, panCK, CD21, CD4, CD8, and CD20. Public datasets compared TLS signatures between NHT-treated and NHT-naïve patients, and between paired pre-/post-NHT samples. An orthotopic immunocompetent PCa mouse model was generated using organoids and CRISPR-Cas9. Flow cytometry evaluated immune infiltration in bicalutamide-treated mice, and anti-PD1 was combined with degarelix/bicalutamide in preclinical mouse model.

**Results:**

TLS were detected in 93% of NHT-naïve PCa tissues, predominantly within tumors. Mature TLS, secondary follicle-like TLS (SFL-TLS), and higher intra-tumoral TLS density correlated with prolonged progression-free survival (PFS). NHT-treated patients exhibited elevated TLS maturity, density, and immune infiltration (CD4+, CD8+, CD20+, CD21+ cells). Matched biopsies confirmed NHT enhanced TLS detection, maturation, and intra-TLS CD8+ T cell infiltration. Transcriptomics revealed upregulated CD4, CD8, CD20, and FOXP3 post-NHT. In mice, androgen deprivation therapy (ADT) increased immune infiltration, and anti-PD1/ADT combination improved tumor response.

**Conclusion:**

Our findings demonstrate that TLS serve as a favorable prognostic biomarker in prostate cancer. NHT enhances TLS formation, maturation, and immune cell infiltration, suggesting a synergistic role for androgen deprivation in shaping the tumor immune microenvironment. The improved anti-tumor response with combined anti-PD1 and ADT highlights the potential of immunotherapy-endocrine therapy combinations as a promising treatment strategy for PCa.

## Introduction

Prostate cancer (PCa) is a prevalent malignancy with rising global incidence and mortality (1, 2). First-line treatments, including prostatectomy and androgen deprivation therapy (ADT), are often effective initially, but many advanced cases progress to lethal metastatic castration-resistant prostate cancer (mCRPC) within an immunosuppressive tumor microenvironment (TME) (3–5). Immune checkpoint blockade (ICB) therapies, though effective in other cancers, show limited success in PCa, primarily due to its low lymphocyte infiltration, classifying it as a ‘cold tumor’ (6–8). Developing strategies to convert cold tumors into hot tumors is essential for enhancing antitumor immunity and achieving durable therapeutic responses. Studies have investigated how various factors and therapeutic interventions alter the immune microenvironment in prostate cancer. Dallos et al. demonstrated that ADT could transformed the immune phenotype into an inflamed environment in PCa, characterized by increasing activated CD8 T cells and proinflammatory M1 tumor-associated macrophages (9). However, the relationship between tertiary lymphoid structures (TLS), a critical immune marker, and prostate cancer remains largely unexplored.

TLS refers to the aggregation structure of immune cells (mostly T and B cells) located in non-lymphoid tissues (10). Previous studies indicates that the presence of TLS have varying impacts on prognosis depending on the cancer type (11–13). TLS is associated with better outcomes in multiple types of cancer, including lung, colorectal, and pancreatic cancer (10). Patients with higher levels of TLS were observed to have prolonged overall survival (OS) and disease-free survival (DFS), and TLSs presence was reported to be a favorable prognostic marker (14, 15). This relationship is partly due to TLS’s role in facilitating local immune responses, which can enhance anti-tumor activity in the tumor microenvironment (16). TLS can promote a protective antitumor immune response regulated by T cells in prospective and retrospective non-small cell lung cancer (NSCLC) patient (17). An ex vivo patient-derived tumor fragment platform, including melanoma, breast, lung or renal cancers, demonstrated that the presence of TLS correlates with the capacity for intratumoral immune cell reactivation in response to PD-1 blockade (18). In contrast, in some other types of cancer, the presence of TLS was reported to be associated with worse survival outcomes. Hepatocellular carcinoma (HCC) containing immature TLS exhibited a high expression of gene involved in immunosuppression and immune cell exhaustion (19). In intrahepatic cholangiocarcinoma (iCCA) patient, peri-tumor TLS was associated with worse survival (20). The prognostic significance of TLS in cancer remains debated, with conflicting findings. Nevertheless, most evidence supports their role as biomarkers for predicting immunotherapy response (21, 22). Cancer treatments can reshape the TME, often driving sustained lymphocyte infiltration through the induction of TLS. These structures amplify both T- and B-cell responses and have emerged as strong predictive markers for the efficacy of anti-PD-1 and anti-CTLA-4 therapies across multiple cancers (21). Recent research has focused on inducing TLS formation and maturation to enhance immunotherapy efficacy and improve cancer treatment outcomes (23, 24).

Few studies have examined TLS in prostate cancer, reporting their presence, immune composition, and association with favorable outcomes. TLS have been detected across different stages of PCa, primarily composed of Tbet^+^ T cells and CD8^+^ T cells (25). Another study confirmed TLS presence in PCa, reporting positive correlations with MHC expression as well as T- and B-cell cluster signatures (26). Importantly, mature TLS in PCa were associated with improved clinical outcomes and a more immunologically active TME (27). While the available data are limited, they provide a valuable basis. Building on this, our study further investigates TLS characteristics in PCa and their relationship with neoadjuvant hormone therapy (NHT). In this study, we comprehensively examined the characteristics of TLSs in PCa, inter-patient TLSs heterogeneity and its correlation with clinical outcomes. We assessed prostate cancer tumor samples and observed marked variation in TLS spatial distribution, density, and maturation across patients. The presence of mature TLS was associated with prolonged progression-free survival. Transcriptomic analysis further revealed that patients receiving NHT showed higher immune infiltration and stronger TLS signatures compared with untreated patients. To provide direct evidence of NHT effects on TLS formation, we analyzed paired prostate biopsy and prostatectomy specimens. Pathological evaluation demonstrated an increased frequency and maturation of TLS after NHT, accompanied by enhanced T-cell infiltration, particularly in bicalutamide-treated tumors. These findings were validated in a primary orthotopic prostate cancer mouse model, where androgen deprivation therapy (ADT) remodeled the TME and improved antitumor responses when combined with immunotherapy. Together, our results indicate that TLS serve as favorable prognostic biomarkers in prostate cancer. Moreover, NHT promotes TLS formation and maturation, thereby fostering a more immunologically active TME.

## Materials and methods

### Patients

**Figure 1:**
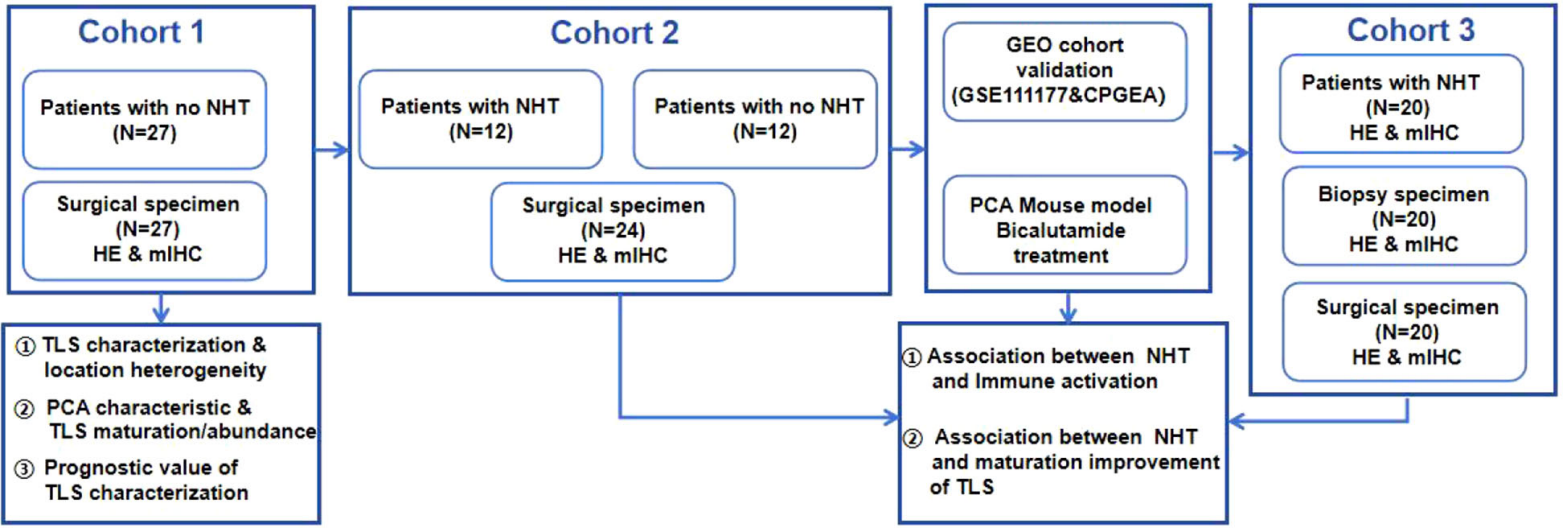
Overall study design.

Five independent cohorts comprising a total of 109 PCa patients were collected. Between 1 July 2020 and 20 January 2022, 71 patients diagnosed with prostate cancer were enrolled in the department of urology, Xx Hospital. As shown in **Figure 1**: Cohort 1: 27 patients without NHT treatment; Cohort 2: 12 patients with NHT treatment and 12 patients without NHT treatment; Cohort 3: 20 patients with NHT treatment enrolled. All patients received prostate biopsy and underwent radical prostatectomy. Two public dataset PCa cohorts including GSE111177 and CPGEA cohort were also collected for bioinformatic validation. GSE111177 comprised pre-ADT and post-ADT samples from 24 prostate cancer patients (28). CPGEA cohort comprised 134 Chinese PCa patients (7 with NHT and 127 without NHT) (29). This study is in compliance with the 1964 Declaration of Helsinki and is approved by the Ethics Committee. All participants signed informed consent forms.

### H&E and multi-color immunohistochemistry

Tumor sections of 5 mm thickness were cut from tumor tissues fixed by 4% paraformaldehyde. Biopsy slides were obtained from the pathology department of Xx Hospital, which are sequential slides from pathologically diagnosed tumor positive samples. H&E staining was performed according to the standard protocol using hematoxylin and eosin. For mIHC, tissue slides were deparaffinized with xylene and rehydrated through a graded series of ethanol solutions (100%, 95% and 70%). Then, slides were treated by microwave to induce antigen retrieval using citric acid solution for 15 min. 6 primary antibodies were used, including CD4 (1:500, #Ab67480, Abcam), CD8 (1:300, #66868-1-Ig, proteintech), CD20 (1:1000, #60271-1-Ig, proteintech), CD21 (1:200, #24374-1-AP, proteintech), Ki67(1:1000, #HA721115, HuaBio), pan-cytokeratin (1:2000, #HA601094, HuaBio), DAPI (1:500, #C1002, Beyotime). Antibody information was listed in **Supplementary Table 2**.

The slides were then incubated with secondary antibodies (1:1,100 μl for each slide; HRP-anti-rabbit IgG, ZSGB, PV-6001; or HRP-anti-mouse IgG, ZSGB, PV-6002) for 10 min at room temperature. After each cycle of staining, heat-induced epitope retrieval was performed to remove all the antibodies including primary antibodies and secondary antibodies. Multiplex immunofluorescence staining was performed using the AlphaTSA Multiplex IHC Kit (AXT36100031, AlphaX) and IRIS Kit HyperView mIF kit (LuminIris; MH010101). The samples were counterstained for nuclei with DAPI for 10 min and mounted in mounting medium. Multispectral images were scanned with ZEISS AXIOSCAN 7. Cells of interest were quantified using QuPath (v.0.2.0).

### Bioinformatic analysis

The RNA-seq data of GSE111177 produced by the Illumina HiSeq platform was retrieved from GEO database (https://www.ncbi.nlm.nih.gov/geo/query/acc.cgi?acc=GSE111177) and normalized as transcripts per million (TPM). R package “edgeR” was used to explore the differentially expressed genes (DEGs) between pre-ADT and post-ADT groups. In the present study, we considered genes with adjust P value < 0.05 and |log2 fold change (FC)| ≥ 2.0 as DEGs. 210 up-regulated genes and 317 down-regulated genes were screened. After identifying DEGs, we conducted gene ontology (GO) enrichment including Biological Process (BPs) and Kyoto Encyclopedia of Genes and Genomes (KEGG) pathway analysis by R package “clusterProfiler” to explore potential functions of DEGs. Gene sets and KEGG signaling pathways with P value < 0.05 were thought to be significantly enriched. The RNA sequencing data of Chinese Prostate Cancer Genome and Epigenome Atlas (CPGEA) cohort enrolled in this study is downloaded from the CPGEA database (https://github.com/nationstrong/CPGEA), derived from 208 paired tumor tissue samples which were matched with healthy control tissues. Only 134 tumor samples with NHT information (7 with NHT and 127 without NHT) were provided, we normalized RNA-seq data of CPGEA as transcripts per million (TPM). R package “edgeR” was also used to explore the differentially expressed genes (DEGs) between NHT and with-ADT groups. The immune expression levels of the 28 immune cells and four TLS signatures were quantified by single sample gene set enrichment analysis (ssGSEA) via R package “GSVA” (30).

### Mice

Mice were kept in the specific pathogen-free animal facility in Xx University with autoclaved food, bedding and water. Animals were housed at room temperature (23 ± 2 °C) at a humidity of 30–70% on a 12-h light/12-h dark cycle (6:00–18:00). All mouse experiments were performed in compliance with the Guide for the Care and Use of Laboratory Animals of Xx University and were approved by the Animal Care and Use Committee of Xx University. C57BL/6 (Jackson Laboratories, 000664) and CAG-Cas9-EGFP mice (Jackson Laboratories, 026179) (male, 8–10 weeks old and ~20 g weight) were used. Mice were monitored for tumorigenesis by bioluminescent imaging. In our mouse experiments, the tumor volumes did not exceed the maximal permitted tumor volume of 1,000 mm^3^. The work has been reported in accordance with the ARRIVE guidelines (31).

### Cell culture

HEK 293T cells (CRL-1573) were from ATCC and were cultured at 37 °C with 5% CO_2_ in DMEM supplemented with 10% (vol/vol) fetal bovine serum and penicillin (100 U ml–1)/streptomycin (0.1 mg ml–1). The HEK 293T cell line was routinely tested for mycoplasma by PCR. Experiments were performed within 4 weeks after fresh viable cells were thawed.

### Prostate organoid culture

Mouse prostate were removed, flushed, cut into 5 mm^3^ pieces and incubated with digestion buffer containing 1.0 mg ml^–1^ collagenase II(YEASEN, 40508es76) in DMEM/F12 (Gibco, C11330500BT) for 40 minutes at 37 °C, mechanical pipetting every 15 minutes, followed by filtration through 100 mm cell strainers, collected by centrifugation and resuspended in ice-cold Matrigel (Corning, Cat# 356237). The mixture was plated into a 48-well tissue culture plate (30 uL drop with 10,000 cells) and incubated for 15 min at 37 °C. Pre-warmed organoid culture medium was then added. The basic culture medium for mouse prostate organoids was slightly modified from a previous report (32), where DMEM/F12 was supplemented with penicillin/streptomycin (Gibco, 15140-122), 1× B27 (Gibco, A3582801), 1 mM N-acetylcysteine (Sigma, A9165), 10 uM Y-27632 dihydrochloride, 1nM DHT (Aladdin, D413176), 50 ng ml^–1^ mouse recombinant epidermal growth factor (Peprotech, AF-100-15-1000), 100 ng ml^–1^ mouse recombinant noggin (Peprotech, 120-10C-250), 10% R-spondin conditioned medium and 500 nM A83-01 (Peprotech, 9094360).

For organoid passing, prostate organoids were released in TrypLE (Gibco, 12605-028), and mechanical dissociation was performed every 5 minutes at 37 °C, followed by centrifugation at 1,500 rpm for 5 minutes. Ultimately, single cells were reseeded in Matrigel and cultured as described above. The organoid medium was refreshed every 2–3 d.

### Organoid genome editing

sgRNAs designed on the CRISPR Design Tool (https://www.benchling.com/crispr) were cloned into the lentiviral vector V2TC which expressed sgRNA and mCherrry. sgRNA sequences were list in **Supplementary Table 3**. Organoids were dissociated using TrypLE (Gibco, 12605-028) and cells were mixed with lentivirus and centrifuged for 1 hr at 2,000 rpm, and then incubated for 1.5 hr at 37 °C, finally resuspended with ice-cold Matrigel. Mutation validation was performed by the T7E1 (Vazyme, Cat# EN303-01) assay.

### Organoid orthotopic transplantation

The primary orthotopic prostate cancer model was generated by injecting mouse normal prostate organoids with Trp53, Pten and RB1 mutation as well as Myc amplification into the prostate. Gene edited organoids were digested with TrypLE at 37 °C for 10 minutes and spun at 400g for 5 min at room temperature. The collected cells were resuspended with 50% Matrigel mixed with PBS. Organoid suspension was injected into the prostate of C57B/L6 mice (male, 8 weeks) using 29-gauge insulin syringe after anesthesia induction.

### Bioluminescent imaging

For bioluminescent imaging, mice were given 200 uL (150 mg/kg) D-luciferin potassium salt (YEASEN, 40902ES03) intraperitoneally and imaged on the IVIS Spectrum *In Vivo* Imaging System (PerkinElmer IVIS Lumina Series III).

### *In vivo* treatment

When luminescent value of the orthotopic tumors reached 1.0*10^7^, the mice were allocated into two different treatment groups: Vehicle control (0.9% saline) and bicalutamide group (MCE, HY-14249) every 3 days by intraperitoneal injection for two weeks. For combination treatment experiment, 12 mice were divided into four different groups: vehicle control (0.9% saline), ADT group (degarelix, 0.625mg/mouse, every 28 days, s.c, MCE, HY-16168A; bicalutamide, every 3 days, intraperitoneal injection, MCE, HY-14249), immunotherapy group (PD-1 blockade, 200ug/mouse, intraperitoneal injection on days 0, 4, 8, 12, 16, 21, selleck, A2122). Immunotherapy combined with ADT group. The treatment started when the luminescence value of the orthotopic prostate tumor reached 10^8^ and lasted for 3 weeks.

### Flow cytometry

Total cells were obtained from single-cell suspensions from the mice orthotopic prostate tumor, after lysis of red blood cells, stained with fluorochrome-conjugated mouse anti- bodies raised against specific markers in PBS. Antibody information is listed in **Supplementary Table 2**.

Flow cytometry analyses were conducted on the Agilent NovoSampler Q and data were analyzed using FlowJo Version 10.8.1 software (Ashland, OR).

### Statistical analysis

Two-group comparisons utilized unpaired Student’s t-tests for normally distributed variables and the Wilcoxon rank-sum test for non-normally distributed variables. The log-rank test was utilized to compare survival differences among groups, specifically for the Kaplan-Meier survival curve analysis. Box plots and bar plots were generated using the ggpubr (v0.5.0) package.

## Results

### Spatial heterogeneity and clinicopathological relevance of TLS maturation and abundance in prostate cancer

**Figure 2:**
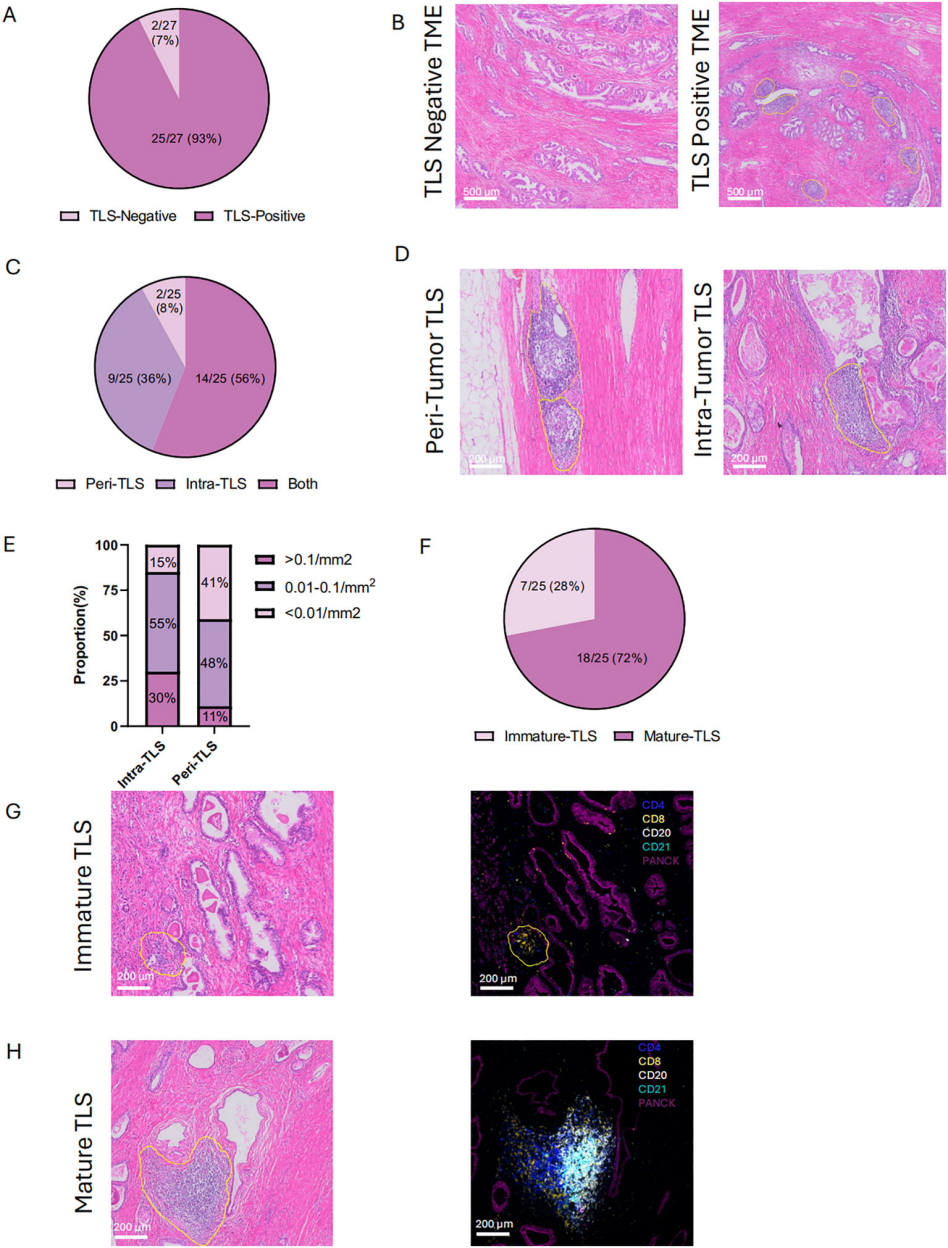
Location and maturation status of tertiary lymphoid structures (TLS) **(A, B)** Histological comparison of TLS-negative and TLS-positive in cohort 1. **(C, D)** Distribution and of histological representation of peri- and intra-tumoral TLS in cohort 1.**(E)** Density of TLS in peri- and intra-tumoral regions. **(F)** Proportion of immature and mature TLS in cohort 1. **(G,H)** HE and mIHC of immature and mature TLS. Peri- tumoral TLS: TLS which located at the leading edge of carcinoma with benign prostatic tissue at one end. Intra- tumoral TLS: TLSs which were surrounded on all sides by tumor. Immature TLS: specific aggregations of lymphocytes. Mature TLS: germinal center B cells localize in the central area, with CD4+ and CD8+ T lymphocytes distributed in the peripheral regions. Statistical significance was determined by Wilcoxon rank-sum test **(B-E)**.

Surgical specimens from cohort 1 patients were collected, and the baseline characteristics of these patients were included in **Supplementary Table 1**. H&E staining of diagnostic sample was used to preliminarily analyze the presence, localization (intra-tumor or peri-tumor) and maturation of TLS (**Figure 2A**). Lymphoid aggregate clusters were firstly identified in 25 (93%) patients (**Figure 2B**). As for the location of TLS, intra TLSs were defined as TLSs which were surrounded on all sides by tumor and peri-TLSs were defined as TLS which located at the leading edge of carcinoma with benign prostatic tissue at one end (**Figure 2C**). Among 25 patients with TLS, 14 (56%) exhibited both intra-TLS and peri-TLS, while intra-TLS and peri-TLS were found exclusively in 9 (36%) and 2 (8%) of patients, respectively (**Figure 2D**). The number of TLSs detected were divided by the tumor area to represent its density. The median TLS density was 0.1/mm2 and the median intra- TLS median density was 0.04/mm2. Using this cut-off value of 0.01/mm2 and 0.1/mm2, the intra-TLS and peri-TLS in 27 patients were classified (including TLS-negative) according to the TLS density (**Figure 2E**).

To further confirm the structures of TLSs, we identified lymphocyte cell clusters with a given marker through mIHC-stained sessions. Immature TLSs normally identified as specific aggregation of lymphocytes (**Figure 2G**), while in mature TLSs, germinal center B cells were located in the central area with CD4+ cells and CD8+ T lymphocytes scattered in the peripheral regions (**Figure 2H**). Mature TLSs could be further divided into Primary Follicle-like Tertiary Lymphoid Structure (PFL-TLS) and Secondary Follicle-like Tertiary Lymphoid Structure (SFL-TLS). In SFL-TLS, CD21+ follicular B cells were intensely clustered in core germinal center (GC) (**Supplementary Figure 1**). **Supplementary Figure 5A** showed the distribution of individual data points in Cohort 1. After analysis of clinicopathological characteristics, the correlation between PCa stage and maturation of TLS was identified (**Table 1**). Both univariate and multivariate regression analyses identified the Gleason score as an independent predictor for SFL-TLS formation (univariate analysis: P = 0.039, HR, 0.143, 95% CI, 0.022 to 0.910; multiunivariate analysis: P = 0.046, HR, 0.126, 95% CI, 0.016 to 0.967). While other variables including age, PSA level, and T stage did not demonstrate significant association with TLS density or maturation.

**Table 1:** Univariate and multivariate analysis of clinical characteristics for TLS formation.

TLS Maturation				
	Univariate analysis		Multivariate analysis	
Characteristics	OR (95% CI)	P value	OR (95% CI)	P value
Age ≤70 or >70	1.600 (0.302, 8.490)	0.581	/	/
PSA ≤10 or>10	0.080 (0.008, 0.781)	0.030	0.104 (0.010, 1.085)	0.059
T stage 2 or 3a	1.600 (0.302, 8.490)	0.581	/	/
Gleason ≤3+4 or ≥4+3	0.182 (0.029,1.139)	0.069	0.272 (0.037, 2.017)	0.203
SFL-TLS Presence				
	Univariate analysis		Multivariate analysis	
Characteristics	OR (95% CI)	P value	OR (95% CI)	P value
Age ≤70 or >70	5.200 (0.924, 29.260)	0.061	5.980 (0.839, 42.615)	0.074
PSA ≤10 or>10	0.250 (0.046, 1.365)	0.109	/	/
T stage 2 or 3a	1.257 (0.249, 6.357)	0.782	/	/
Gleason ≤3+4 or ≥4+3	0.143 (0.022, 0.910)	0.039	0.126 (0.016, 0.967)	0.046
TLS Density				
	Univariate analysis		Multivariate analysis	
Characteristics	OR (95% CI)	P value	Multivariate analysis OR	P value
Age ≤70 or >70	2.640 (0.539, 12.938)	0.231	/	/
PSA ≤10 or>10	0.500 (0.105, 2.379)	0.384	/	/
T stage 2 or 3a	2.640 (0.539, 12.938)	0.231	/	/
Gleason ≤3+4 or ≥4+3	0.648 (0.138, 3.036)	0.582	/	/

### Association between TLS characteristics and PCa prognosis

**Figure 3:**
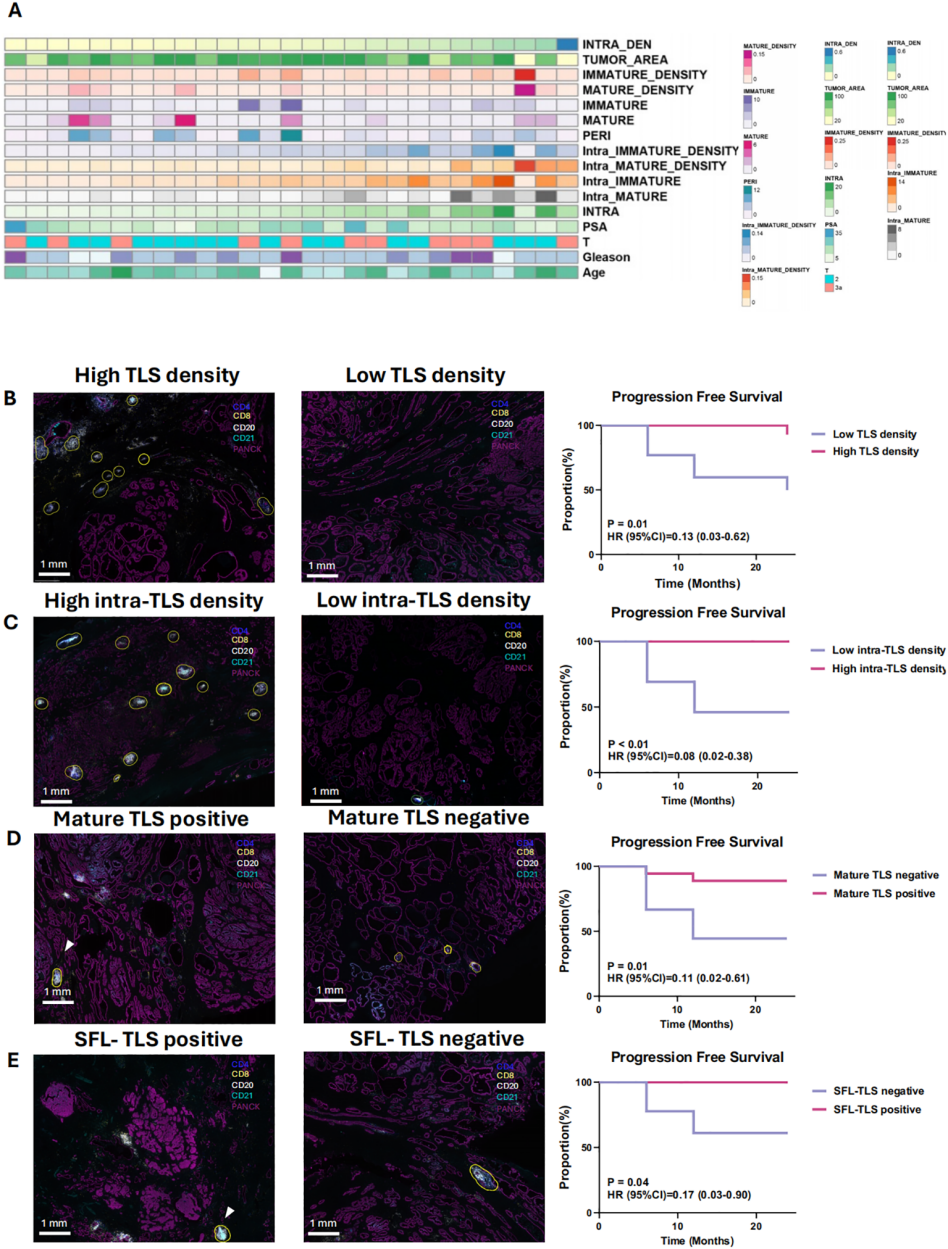
Clinicopathological features and prognostic significance of tertiary lymphoid structures (TLS) **(A)** The distribution of clinical features and immature density for the 27 patients in cohort 1. **(B)** Kaplan-Meier survival curves for progression-free survival (PFS) of 27 patients with no treatment before surgery stratified based on the median density of TLSs **(B)**, density of intra-/peri- TLSs **(C)**, maturation of TLSs **(D)** and the existence of SFL-TLSs (In SFL-TLS, CD21+ follicular dendritic cells form dense clusters within the core germinal center) **(E)**. B–E: Differentiate Kaplan-Meier curves with distinct colors for better visual clarity. 3D & 3E: Clarify the difference between “mature TLS” and “SFL-TLS.”.

In this study, follow-up records (median follow-up period of >12 months) of 27 patients in cohort 1 were retrospectively included. The baseline clinicopathological characteristics are summarized in **Figure 3A**. Significant prolonged progression free survival was observed in TLS density-high than density-low group (PFS: P = 0.0107, HR = 0.2812). High intra-TLS density was also associated with improved PFS (P = 0.0016, HR = 0.079) (**Figures 3B, C**). As for the correlation between TLSs maturation with the prognosis, patients were divided into mature TLS positive and mature TLS negative group. The presence of mature TLS was associated with improved PFS (PFS: P = 0.0118, HR = 0.111). Similarly, the presence of SFL-TLS was significantly associated with prolonged PFS (PFS: P = 0.0364, HR = 0.1745) as well (**Figures 3D, E**).

### Difference of TLS characteristics between NHT and NHT naive PCa patients

**Figure 4:**
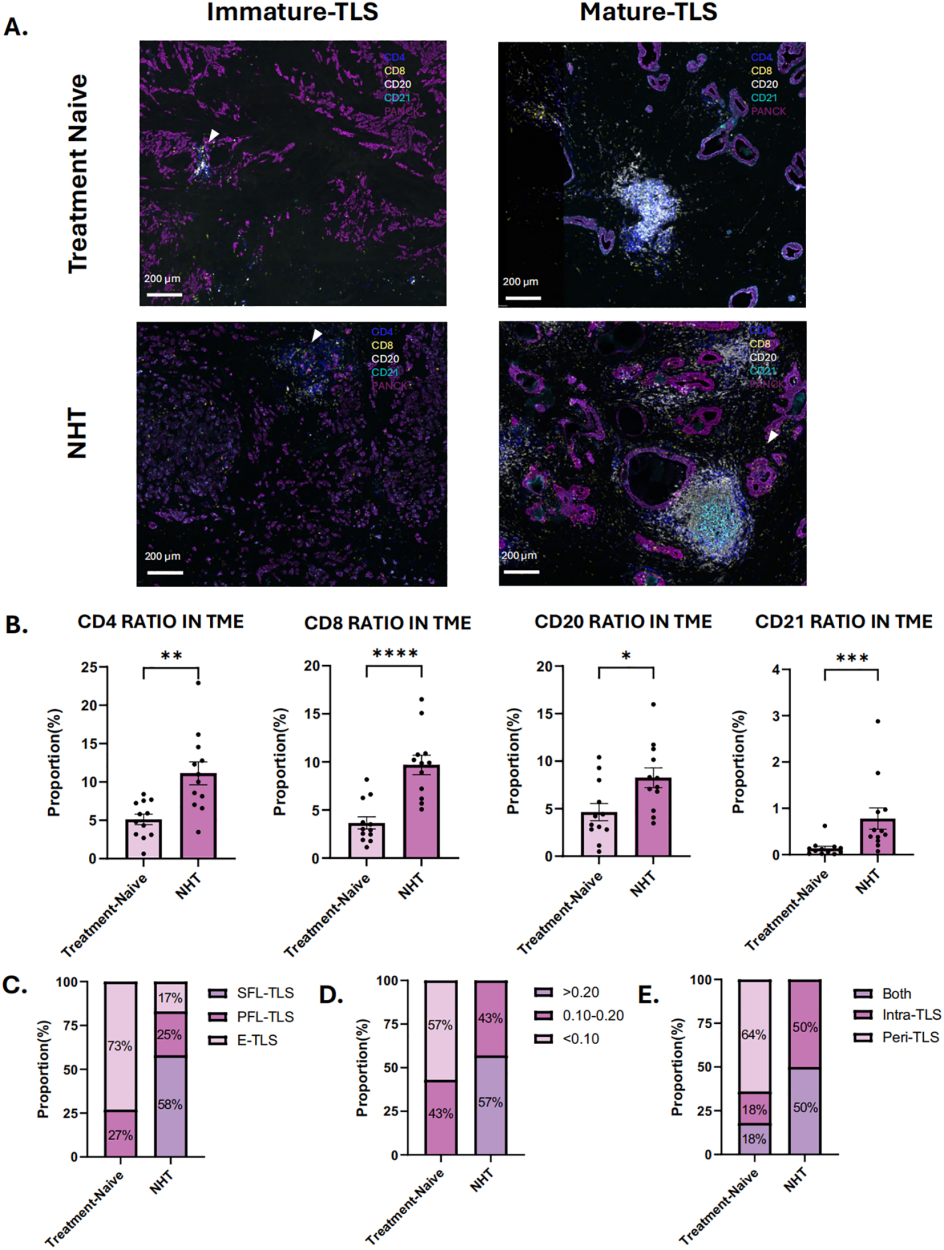
Characteristics of tertiary lymphoid structures (TLS) in treatment-naive group and neoadjuvant hormone therapy (NHT) group in cohort 2. **(A)** mIHC of immature and mature TLS in NHT group and treatment-naive group in cohort 2; **(B)** Comparison of the ratio of CD4 + cells, CD8+ cells, CD20+cells and CD21+cells in tumor microenvironment (TME) in the NHT group and treatment-naive group. **(C)** Proportion of different TLSs maturation stage in the NHT group and treatment-naive group. **(D)** Proportion of different density of TLS in the NHT group and treatment-naive group. **(E)** Proportion of different location of TLS in the NHT group and treatment-naive group. P values are denoted as follows: *p < 0.05, **p < 0.01, ***p < 0.001, ***p < 0.0001.

To further explore the effects of NHT on TLSs formation and maturation, 12 patients who received NHT and 12 patients received no preoperative NHT treatment were prospectively enrolled as cohort 2, their surgical resection specimens were collected for mIHC staining. Baseline characteristics of these patients are summarized in **Supplementary Figure 2A**. **Supplementary Figure 5B** showed the distribution of individual data points in Cohort 2. Morphologically, there is no structural difference of TLSs of each stage between NHT group and NHT treatment-naive group (**Figure 4A** **).** We calculated the infiltration ratio of various immune cells by dividing the number of positive immune cells by the total number of cells in the tissue section. We found CD4, CD8, CD20 and CD21 cell infiltration level were all significantly higher in NHT group (**Figure 4B**). TLSs of different stages were found both in NHT group and treatment naive group. In NHT group, TLS maturation was significantly higher than treatment naive group (P<0.05) (**Figure 4C**). We observed higher TLS density in NHT group (0.20 ± 0.06 vs. 0.08 ± 0.04, P<0.05) (**Figure 4D**). Meanwhile, all samples from NHT group are intra-TLS positive (**Figure 4E**) Thus, we supposed that increased TLS presence and maturity might be a result of NHT intervention.

### Validation of TLS change following NHT by transcriptome analysis

**Figure 5:**
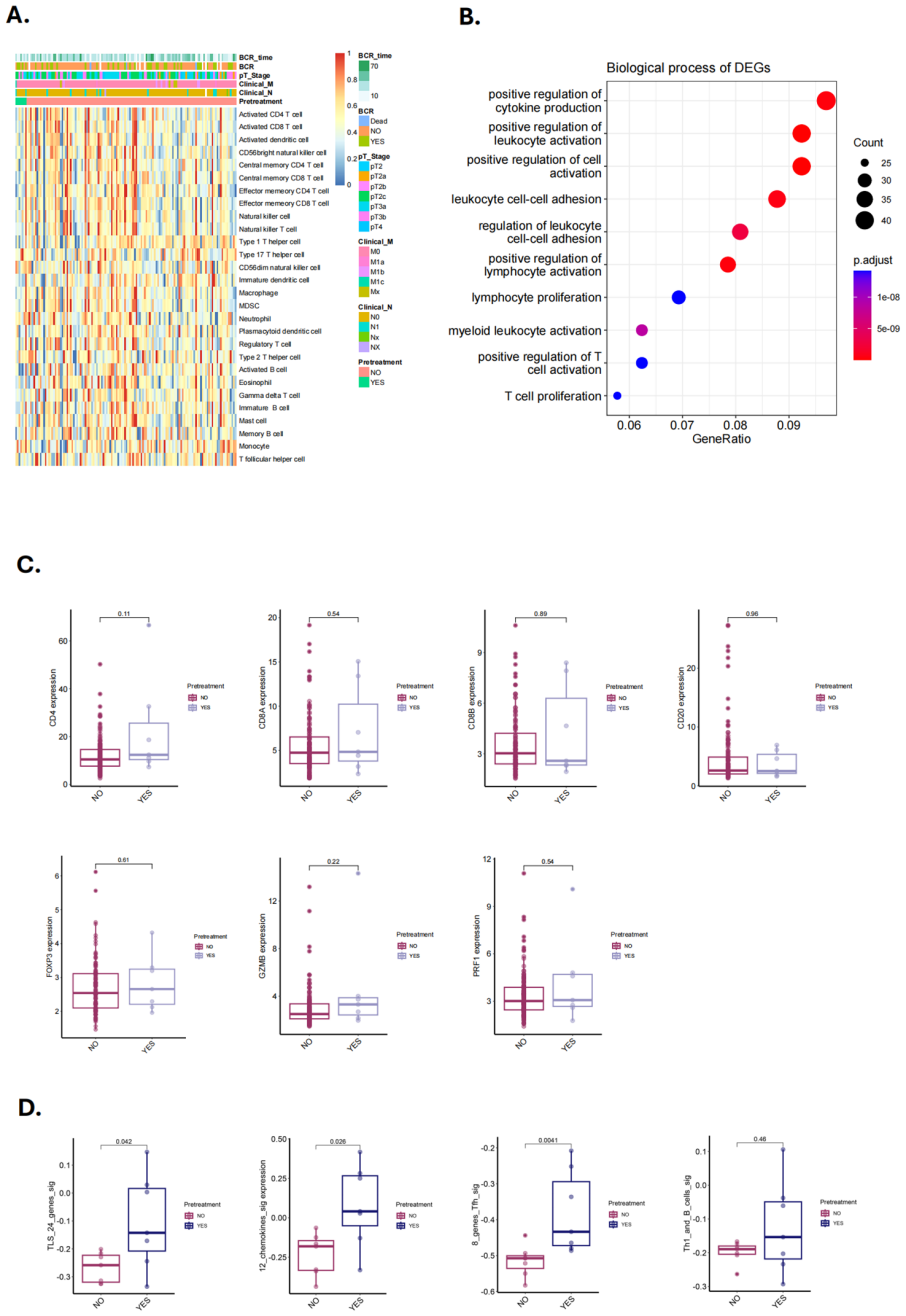
Characteristics and changes of immune cells in with/without neoadjuvant hormone therapy (NHT) groups in CPGEA cohorts. **(A)** Supervised clustering of PCa specimens by NHT (n = 7 with NHT and n = 127 without NHT), displaying ssGSEA scores (CPGEA cohorts). **(B)** The biological process of DEGs identified in with/without NHT groups (CPGEA). **(C)** Comparison of the expression of CD4, CD8A and CD8B, CD20, FOXP3, GZMB, PRF1 in PCa patients with/without NHT using CPGEA cohort (n = 120). **(D)** Comparison of GSVA scores utilizing four TLS signatures in with/without NHT samples from CPGEA cohort.

**Figure 6:**
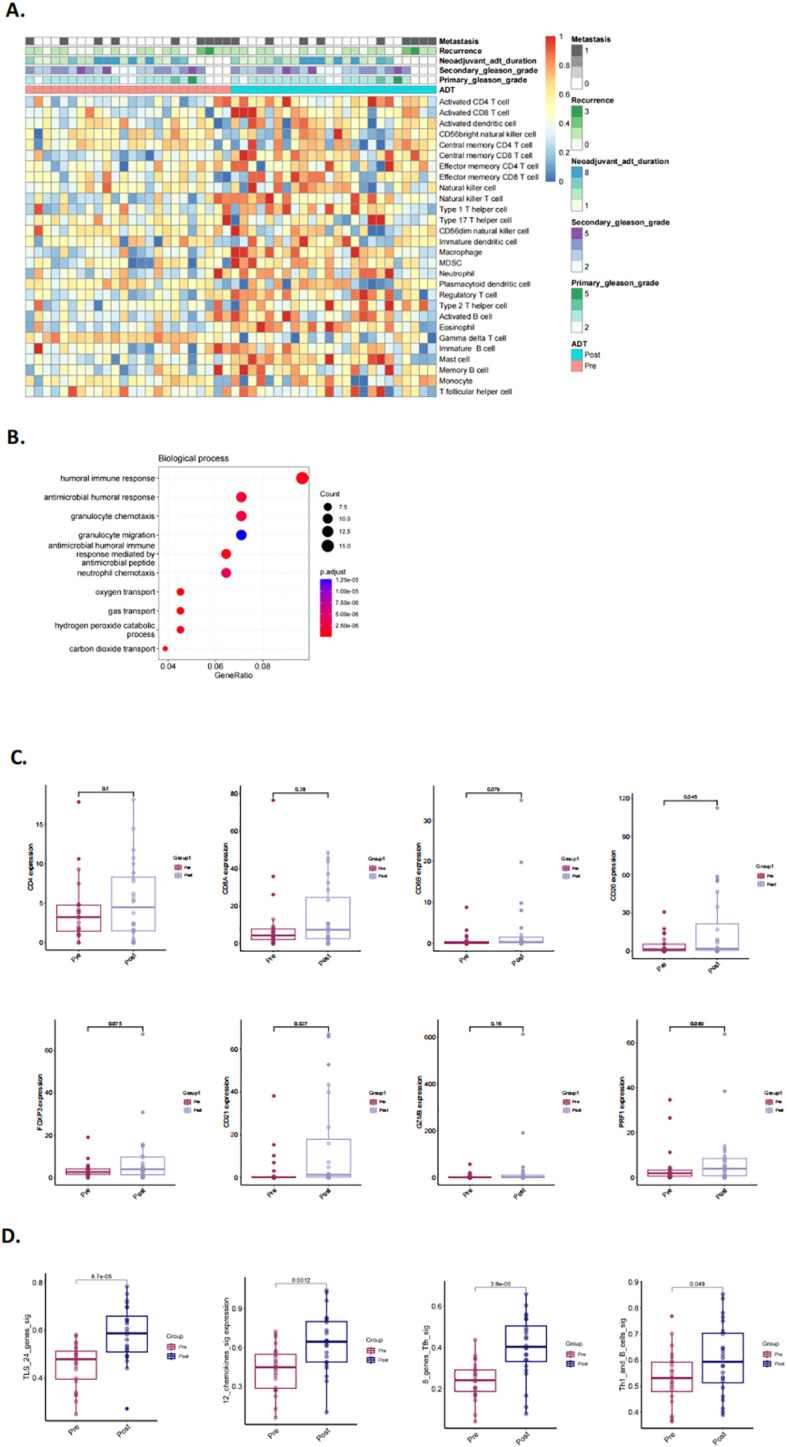
Characteristics and changes of immune cells in pre- and post- neoadjuvant hormone therapy (NHT) groups in GSE111177. **(A)** Supervised clustering of PCa specimens by NHT (n = 24), displaying ssGSEA scores (GSE111177). **(B)** The biological process of DEGs identified in pre-/post- NHT groups (GSE111177). **(C)** Comparison of the expression of CD4, CD8A and CD8B, CD20, FOXP3, CD21, GZMB, PRF1 in PCa patients before and after NHT using GSE111177 cohort (n = 24). **(D)** Comparison of GSVA scores utilizing four TLS signatures in 24 paired pre-NHT and post-NHT samples from GSE111177.

To validate our hypothesis, we utilized two previously published NHT therapy datasets, Cancer Patient Gene Expression Atlas (CPGEA) and GSE111177, for gene set enrichment analysis (GSEA). We then assessed the expression of 28 immune cell gene expression signatures in CPGEA for patients with/without NHT treatment. Gene expression profiling by microarray and subsequent ssGSEA analysis of baseline tumor samples was performed, demonstrating significantly higher expression of immune cells in with NHT group versus without NHT group (**Figure 5A**). We further performed biological process (BP) analysis and found that DEGs between with/without NHT group significantly enriched in cell-cell interaction related pathways (**Figure 5B**). We also explored the expression difference of marker genes, including CD4 for helper T cells, CD8A and CD8B for killer T cells, CD20 for B cells, FOXP3 for regulatory T cells. We also included GZMB and PRF1 to strengthen the identification of cytotoxic T cells (**Figure 5C**). This reveals an upregulation of these marker genes following NHT treatment via CPGEA. We next assessed the expression of 28 immune cell gene expression signatures in 24 paired pre-NHT and post-NHT samples from GSE111177. Gene expression profiling by microarray and subsequent ssGSEA analysis of baseline tumor samples was performed, demonstrating significantly higher expression of immune cells in post-NHT group versus pre-NHT group (**Figure 6A**). 210 up-regulated genes and 317 down-regulated genes were screened (**Supplementary Figure 6**) and BP analysis demonstrated that the DEGs were enriched in immune response related pathways (**Figure 6B**). We also explored the expression difference of marker genes (CD4 for helper T cells, CD8A and CD8B for killer T cells, CD20 for B cells, FOXP3 for regulatory T cells, CD21 for follicular dendritic cells, GZMB and PRF1 for cytotoxic T cells), revealing an upregulation of these marker genes following NHT treatment via GSE111177 (**Figure 6C** **).** In addition, by employing four established TLS gene signatures from the literature, including a 24-gene TLS signature, a 12-chemokine TLS signature, an 8-gene Tfh signature, and a Th1 and B cells signature^24^, we quantified the TLS transcriptional abundance in these datasets. Our analysis consistently revealed an upregulation of these signatures following NHT treatment in both CPGEA (**Figure 5D**) and GSE111177 (**Figure 6D**), which also suggested that NHT treatment might induce TLS formation. We also explored the survival roles of the four gene signatures via 120 patients with follow up information from CPGEA cohort (**Supplementary Figure 2C**), which concluded that patients with higher signature scores had better survival comparing to those with lower TLS signature scores.

### Identification of TLS change following NHT

**Figure 7:**
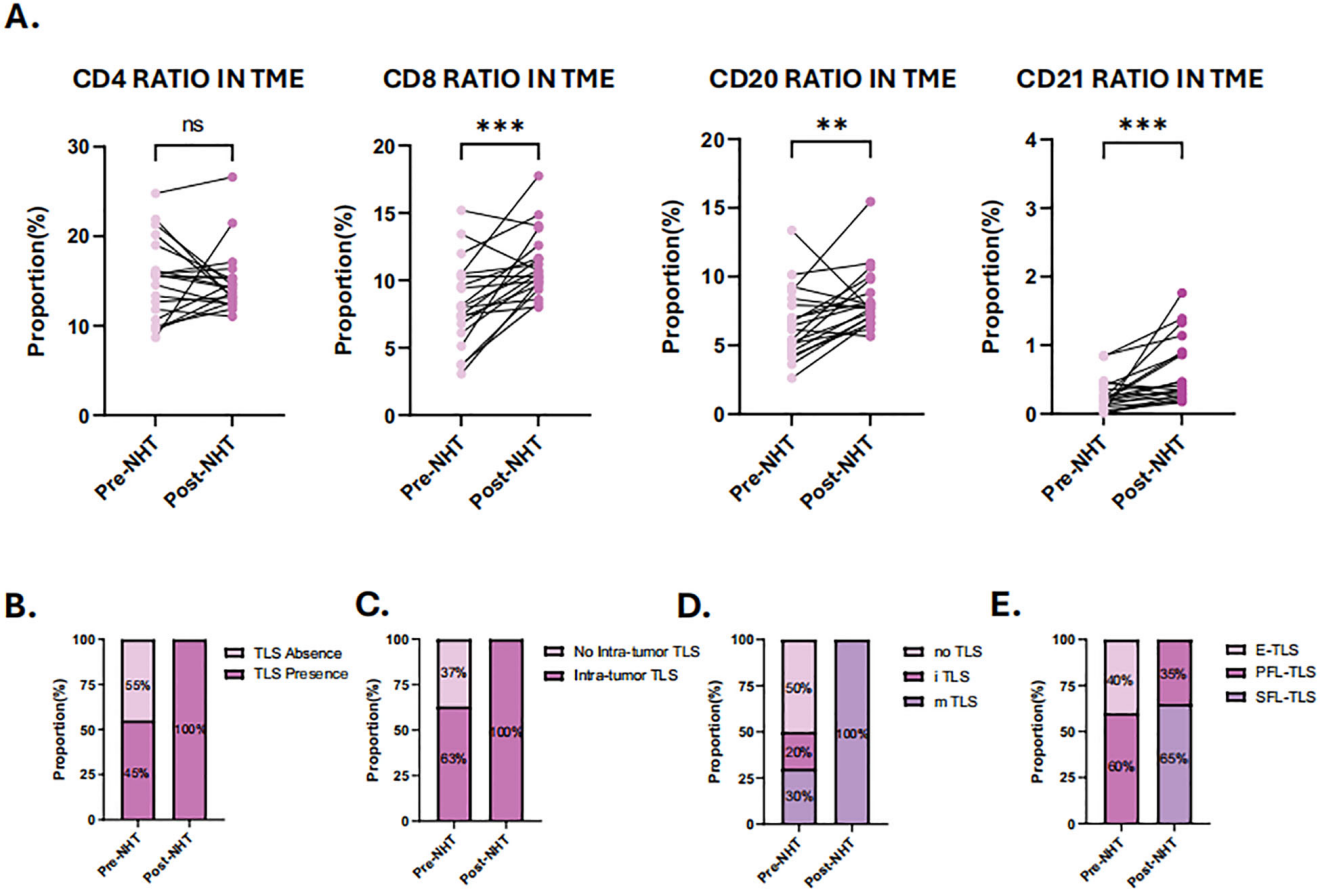
Characterization of tumor immune microenvironment (TME) and tertiary lymphoid structures (TLS) before and after neoadjuvant hormone therapy (NHT). **(A)** Comparison of the ratio of CD4 + cells, CD8+ cells, CD20+cells and CD21+cells in TME before and after NHT. **(B)** Comparison of the TLS presence in TME before and after NHT. **(C)** Comparison of the intra-tumor TLS presence in TME before and after NHT. **(D)** Comparison of the TLS maturation stage in TME before and after NHT. **(E)** Evaluation of TLS maturation stage using mIHC staining of CD4, CD8 CD20, CD21 before and after NHT. P values are denoted as follows: **p < 0.01, ***p < 0.001, NS p>0.05.

To further validate our hypothesis, 20 patients who underwent NHT were prospectively enrolled as cohort 3 and biopsy specimens were analyzed by H&E staining and mIHC (**Supplementary Figure 4A**). All patients were thereafter treated with prostatectomy, and the resected tumor samples were also analyzed by H&E staining and mIHC (**Supplementary Figure 4B**). Baseline characteristics of these patients are summarized in **Supplementary Figure 2B**. **Supplementary Figure 5C** showed the distribution of individual data points in Cohort 3. Before NHT, TLSs were detected in 45% biopsy samples, and mature TLSs were found in 30% biopsy samples. SFL-TLS was not detected in any biopsy sample. E-TLS and PFL-TLS were observed in 40% and 60% TLS positive samples respectively. After NHT, mature TLSs were found in all patients and SFL-TLS was detected in 65% TLS positive samples (**Figures 7B–E**). As for immune infiltration influenced by NHT, the ratio of different immune cells was analyzed in paired pre- and post- NHT samples (**Figure 7A**). Considering the difference in sections area between biopsy tissue and surgical specimen, we calculate the proportion of immune cell number to total cell number in sections to reflect the immune infiltration level. Our analysis revealed a heightened abundance of immune cell infiltration following NHT, as indicated by mIHC. Notably, the infiltration of CD8, CD20 and CD21 cells increased significantly after NHT (**Figure 6A**), which is in consistent with transcriptome analysis of GSE111177 (**Figure 5C**).

### The effect of NHT on PCa tumor infiltrating lymphocytes

**Figure 8:**
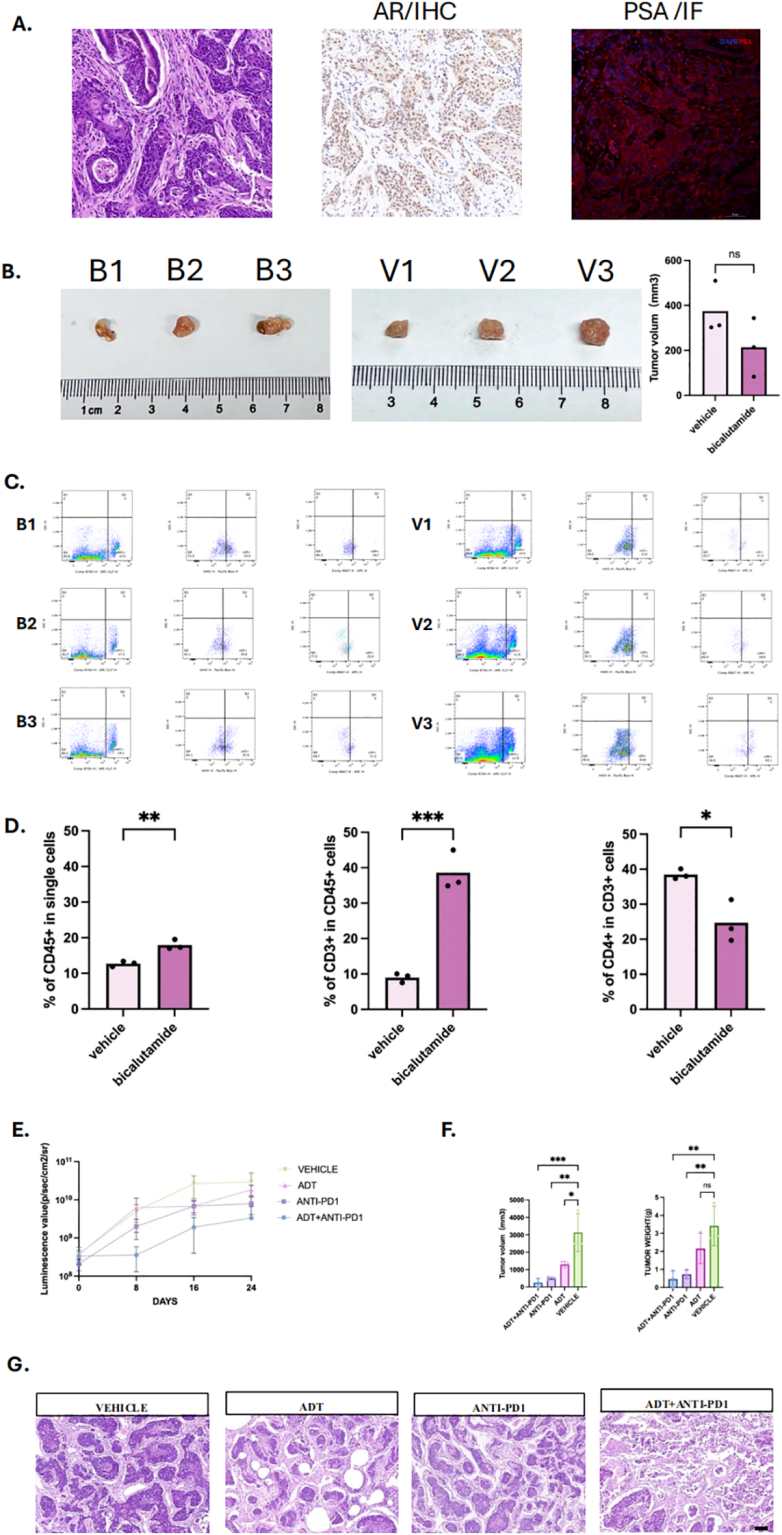
Androgen deprivation therapy enhances immune infiltration and immunotherapy efficacy in prostate cancer mouse model. **(A)** Primary Orthotopic prostate cancer mouse model, including HE staining, with positive immunohistochemistry (IHC) staining for androgen receptor and positive immunofluorescence (IF)staining for Prostate specific androgen (PSA). **(B)** Tumor images of mice in the bicalutamide and vehicle groups, with three mice in each group, labeled as B1, B2, B3 and V1, V2, V3. **(C)** Flow cytometry plots of tumors and infiltrated immune cells from the bicalutamide and control groups. **(D)** Bar graph showing the proportions of CD45+, CD3+, and CD4+ immune cells in tumor tissues from the bicalutamide and control groups. **(E)** Luminescence value of four group: Androgen deprivation therapy (ADT) + immunotherapy (anti-PD1)group, immunotherapy group, ADT group, vehicle group. Measured once a week. **(F)** tumor volume (left) and tumor weight (right) of each four group. **(G)** HE staining for all four groups. P values are denoted as follows: *p < 0.05, **p < 0.01, ***p < 0.001, NS p>0.05.

To validate the relationship between endocrine therapy and immune cell infiltration *in vivo*, we developed a mouse model of primary orthotopic prostate cancer using prostate organoids (**Supplementary Figures 3A, B**). Pathological analysis revealed significant heterogeneity and abnormal glandular structures in the tumor tissues. Immunohistochemical staining confirmed that the tumors were positive for androgen receptors (AR) and prostate-specific antigen (PSA) (**Figure 8A**).

We then treated the mice with bicalutamide. The results showed no significant difference of the mean tumor volume between the bicalutamide-treated group (213.58 mm³) and the control group (374.71 mm³) (**Figure 8B**, P>0.05). However, flow cytometry of the single cells digested from the tumor tissue revealed a higher proportion of CD45+ immune cells in the bicalutamide group compared to the control group (18% vs 12.73%). Additionally, CD45+CD3+ T cells were more abundant in the bicalutamide group (39% vs 9%), while CD45+CD3+CD4+ T cells were fewer in the bicalutamide group (24.65% vs 39.47%) (**Figures 8C, D**). The specified gating strategy was shown in **Supplementary Figure 3**. This finding led us to hypothesize that combining immunotherapy with ADT could enhance the anti-tumor response. We divided the mice into four groups (n=3): the anti-PD1 group, the ADT group, the anti-PD1 combined with ADT group, and the vehicle group. Treatment began when the luminescence value of the orthotopic prostate tumor reached 10^8^. The luminescence values during treatment indicated that the anti-PD1 combined with ADT group exhibited the most effective anti-tumor response (**Figure 8E**). Tumor volume measurements further confirmed the superiority of the combination therapy (**Figure 8F**). Notably, one mouse in the anti-PD1 combined with ADT group showed no detectable tumors after treatment, and their corresponding luminescence values were significantly lower than those of other groups. We further conducted HE staining for each group. Significant necrosis could be observed in the combination therapy group. All three treatment groups exhibited varying degrees of tumor regression (**Figure 8G**).

## Discussion

TLSs are ectopic aggregates of B cells, T cells, and dendritic cells that can support local immune responses. In many tumor types, their presence and maturation correlate with favorable prognosis and improved response to immunotherapy. However, evidence in PCa remains scarce. Our study provides comprehensive data showing that TLSs are present in PCa, display inter-patient heterogeneity, and serve as a favorable prognostic biomarker. Importantly, we demonstrate that NHT promotes TLS formation and maturation, thereby enhancing immune activity in the TME. We observed that mature TLSs correlated with prolonged progression-free survival and were enriched with CD8^+^ T cells and CD20^+^ B cells, suggesting improved antitumor immunity. Transcriptome analysis confirmed stronger immune infiltration and TLS-related gene signatures in NHT-treated patients. Pathological analysis of paired biopsy–prostatectomy samples provided direct evidence that NHT increases both TLS density and maturity.

Consistent with patient data, our orthotopic mouse model revealed that ADT could reshape the immune cell composition within the tumor microenvironment, increasing CD3+ T cell infiltration and a reduction in CD4+ helper T cells, indicating a shift toward CD8+ cytotoxic T cell dominance. Combination therapy with anti-PD1 and ADT exerted a synergistic anti-tumor effect. Remarkably, one mouse in the combination group showed complete tumor regression and significantly lower luminescence values, an outcome not observed in other monotherapy groups. This highlights the translational potential of integrating ADT with immune checkpoint blockade.

While prior studies have explored how NHT alters immune cell infiltration and cytokine responses in PCa, the link between NHT and TLS induction had not been addressed. Our results fill this gap, showing that NHT enhances TLS density, maturity, and CD8+ T cells infiltration within the TLSs, in line with reports from other cancer where modulating the microenvironment facilitates TLS formation and immune cell recruitment (13). Transcriptome analysis also showed upregulation of CD8+ markers, supporting the concept that NHT can convert prostate cancer from a “cold” to a “hotter” state, thereby improving responsiveness to immunotherapy. Previous studies have shown that NHT, particularly when combined with immunotherapy, enhances immune cell infiltration in prostate tumors (33) (34, 35). Our findings extend these observations by suggesting that TLS formation is an additional, critical aspect of immune reprogramming induced by NHT. This indicates that TLS may not only serve as an indicator of immune activation but could also be a key player in mediating the immune response in prostate cancer, especially in the context of ADT. This is supported by our gene set enrichment analysis, which showed upregulation of immune pathways and TLS-associated markers such as CD8 and CD20 in NHT-treated samples.

Although research on TLS and combination therapy in PCa remains limited, our findings align with prior work showing synergy between ADT and immunotherapy. For example, Guan et al. discovered that blocking AR could directly enhancing CD8+ T cell function and prevent T cell exhaustion, increasing IFN-υ expression to further improve immunotherapy response in PCa (36). Another study also revealed that AR inhibited the activity and stemness of male tumor-infiltrating CD8+ T cells by regulating epigenetic and transcriptional differentiation programs. Castration combined with anti-PD-L1 treatment synergistically restricted tumor growth in male mice (37). AR could downregulate MHC I expression to affect antigen presentation. Inhibiting AR improved T cells responses and tumor control (38). Together with our results, these studies suggest that TLS induction may serve as a central mechanism linking ADT to improved immunotherapy responses.

In summary, our work provides clinical and experimental evidence that NHT promotes TLS formation and maturation, enhances CD8^+^ infiltration, and sensitizes tumors to checkpoint blockade. These findings support TLS as both a prognostic biomarker and a mechanistic mediator of ADT-induced immune activation, offering a promising translational strategy for improving outcomes in prostate cancer patients.

### Study limitations and future directions

Despite the robust findings, our study has certain limitations. First, the sample size for some of the analyses, particularly in the matched pre- and post-NHT patient samples, was relatively small, which may limit the generalizability of the results. Future studies with larger cohorts are necessary to validate our findings. Second, patients in the NHT group exhibited statistically higher Gleason scores than No-NHT group in cohort 2. This baseline imbalance may still introduce potential selection bias. Additionally, while we utilized both clinical samples and an orthotopic PCa mouse model, further research is needed to fully elucidate the molecular mechanisms by which NHT promotes TLS formation. Another area for future investigation is the interaction between TLSs and other immune-modulatory treatments, such as immune checkpoint inhibitors (ICIs). Given that TLS presence is associated with improved immune responses, combining NHT with ICIs could provide a synergistic effect, potentially enhancing treatment outcomes in patients with advanced PCa. Studies exploring this combination could pave the way for new therapeutic strategies aimed at leveraging the benefits of TLSs.

## Conclusion

In conclusion, this study highlights the significant role of TLSs in the prognosis of prostate cancer and demonstrates that NHT can modulate the tumor immune microenvironment by promoting TLS formation and maturation. Our clinical data revealed that NHT-treated patients exhibited higher TLS density and maturity, along with increased infiltration of CD4+, CD8+, CD20+, and CD21+ immune cells. *In vivo*, ADT significantly enhanced T cell infiltration and achieved the most effective anti-tumor response when combined with anti-PD1 therapy in prostate cancer mouse model.

These findings underscore the translational potential of integrating ADT with immune checkpoint blockade as a strategy to convert prostate cancer into a more immunologically responsive disease. By leveraging TLS induction and maturation, this combination therapy may overcome the current resistance of prostate cancer to immunotherapy and provide a rational framework for future clinical trials. Further research is warranted to validate these approaches and fully harness the therapeutic value of TLSs in prostate cancer management.

## Data Availability

The original contributions presented in the study are included in the article/**Supplementary Materials**, further inquiries can be directed to the corresponding author/s.

## Supplementary material

Supplementary Figure 1 Immunofluorescence Characterization of Different TLS Maturation Stages in Prostate Cancer Tumor Microenvironment.

Supplementary Figure 2
**(A)** The distribution of clinical features for the 24 patients in cohort 2. **(B)** The distribution of clinical features for the 20 patients in cohort 3. **(C)** Survival analysis of the four TLS signatures via CPGEA cohort.

Supplementary Figure 3
**(A)** Brightfield (BF) images of Normal prostate organoids. **(B)** BF and fluorescent image of CRISPR-Cas9 gene editing prostate organoids(TP53 null,sgPten,sgRb1,c-myc). **(C)** BF images of the prostate tumors in bicalutamide group and vehicle group alongside corresponding fluorescent images. **(D)** Bioluminescent images of mice in bicalutamide group and vehicle group at the start and end of treatment, arranged from left to right as B1, B2, B3, and V1, V2,V3. **(E)** Representative flow cytometry gating strategy. Live cells were first gated, followed by exclusion of doublets to obtain single cells. From this population, CD45^+^ cells were selected, and within them, CD3^+^ T cells were identified. CD4^+^ T cells were then gated from the CD3^+^ population.

Supplementary Figure 4 Immunohistological Analysis of TLS in cohort 3. A: HE and mIHC staining of paired pre- and post-NHT tumor specimens from Patient 1 in cohort 3; B: Comparison of TLS formation and immune marker distribution in paired pre- and post-NHT samples.

Supplementary Figure 5 The distribution of total TLS characteristics in Cohort 1 **(A)**, Cohort 2 **(B)** and Cohort 3 **(C)**. tTLS: total tertiary lymphoid structures number, E_TLS: early TLS, PFL_TLS: primary follicle-like TLS, SFL_TLS: secondary follicle-like TLS, INTRA_number: intra-tumoral TLS number, INTRA_E: intra-tumoral early TLS number, INTRA_P: intra-tumoral primaryTLS number, INTRA_S:: intra-tumoral secondary TLS number. PERI_number: peri-tumoral TLS number, PERI_E: peri-tumoral early TLS number, PERI_P: peri-tumoral primaryTLS number, PERI_S: peri-tumoral secondary TLS number. CD4: Ratio of CD4+ cells in tumor micro-environment. CD8: Ratio of CD8+ cells in tumor micro-environment. CD20: Ratio of CD20+ cells in tumor micro-environment. CD21: Ratio of CD21+ cells in tumor micro-environment.

Supplementary Figure 6 The heatmap of differential gene expression analysis pre- and post- NHT treatment via GSE111177 (**Supplementary Table 4**).
