# Supplementary figures and images for: Characteristics of tertiary lymphoid structures in prostate cancer and the impact of neoadjuvant therapy on their formation and maturation
Source: Front Immunol. 2025 Nov 4;16:1663396. doi: 10.3389/fimmu.2025.1663396 (PMC12623385; doi:10.3389/fimmu.2025.1663396)

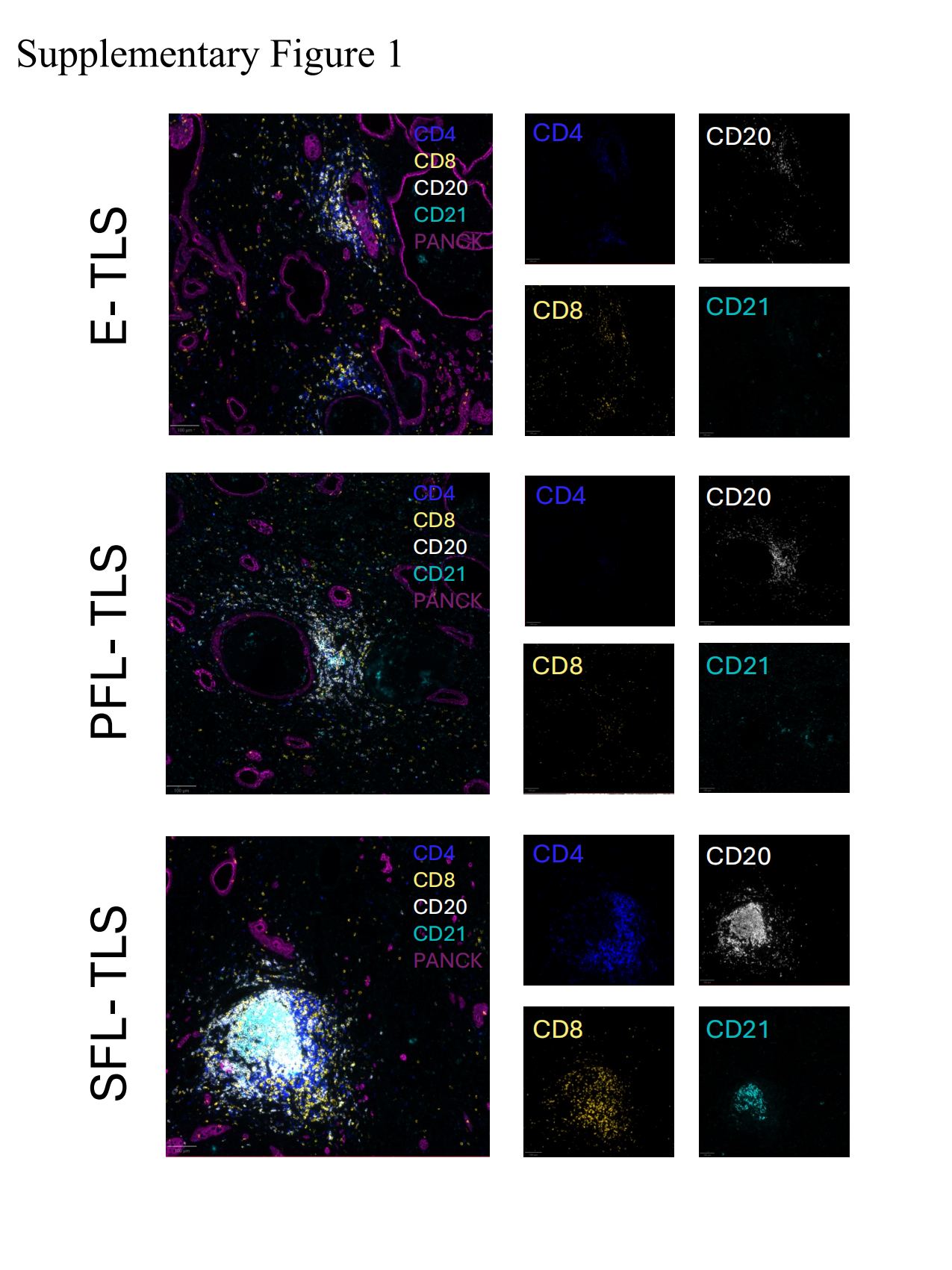

Supplement: Supplementary Figure 1 — Immunofluorescence Characterization of Different TLS Maturation Stages in Prostate Cancer Tumor Microenvironment. [file Image1.tif]

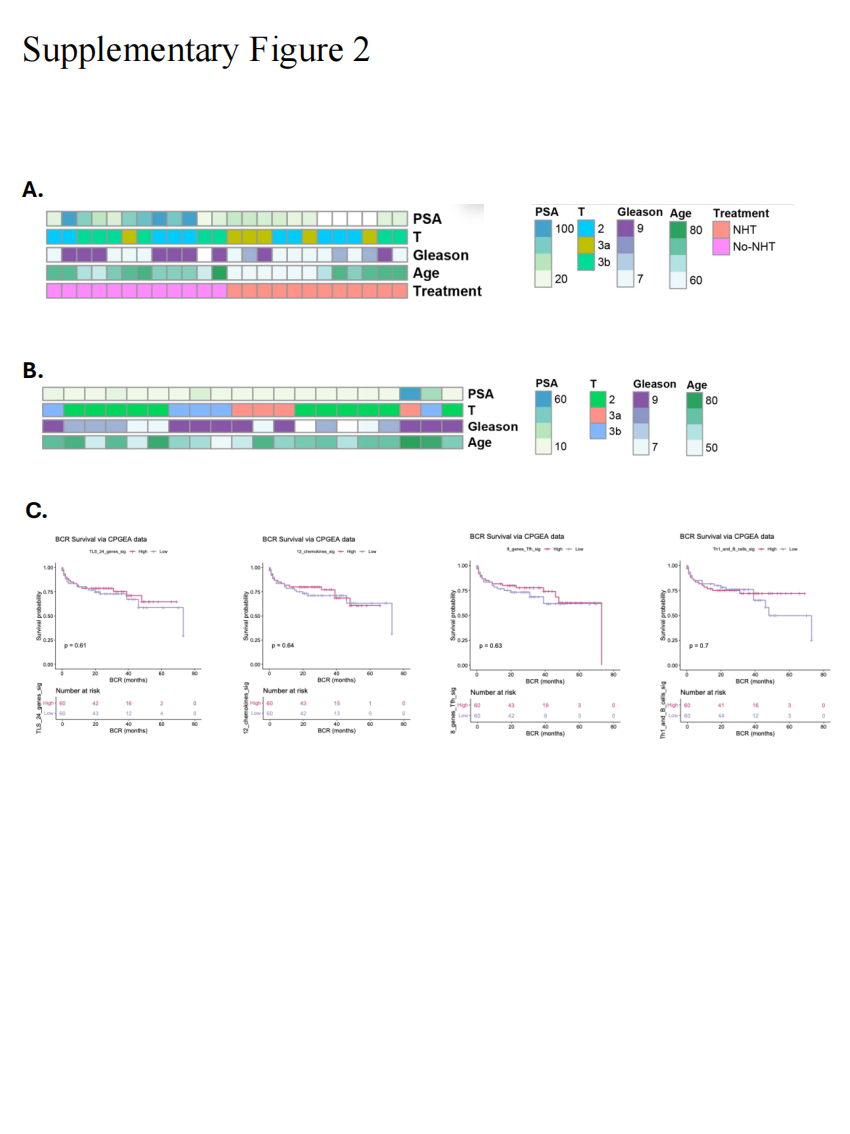

Supplement: Supplementary Figure 2 — (A) The distribution of clinical features for the 24 patients in cohort 2. (B) The distribution of clinical features for the 20 patients in cohort 3. (C) Survival analysis of the four TLS signatures via CPGEA cohort. [file Image2.tif]

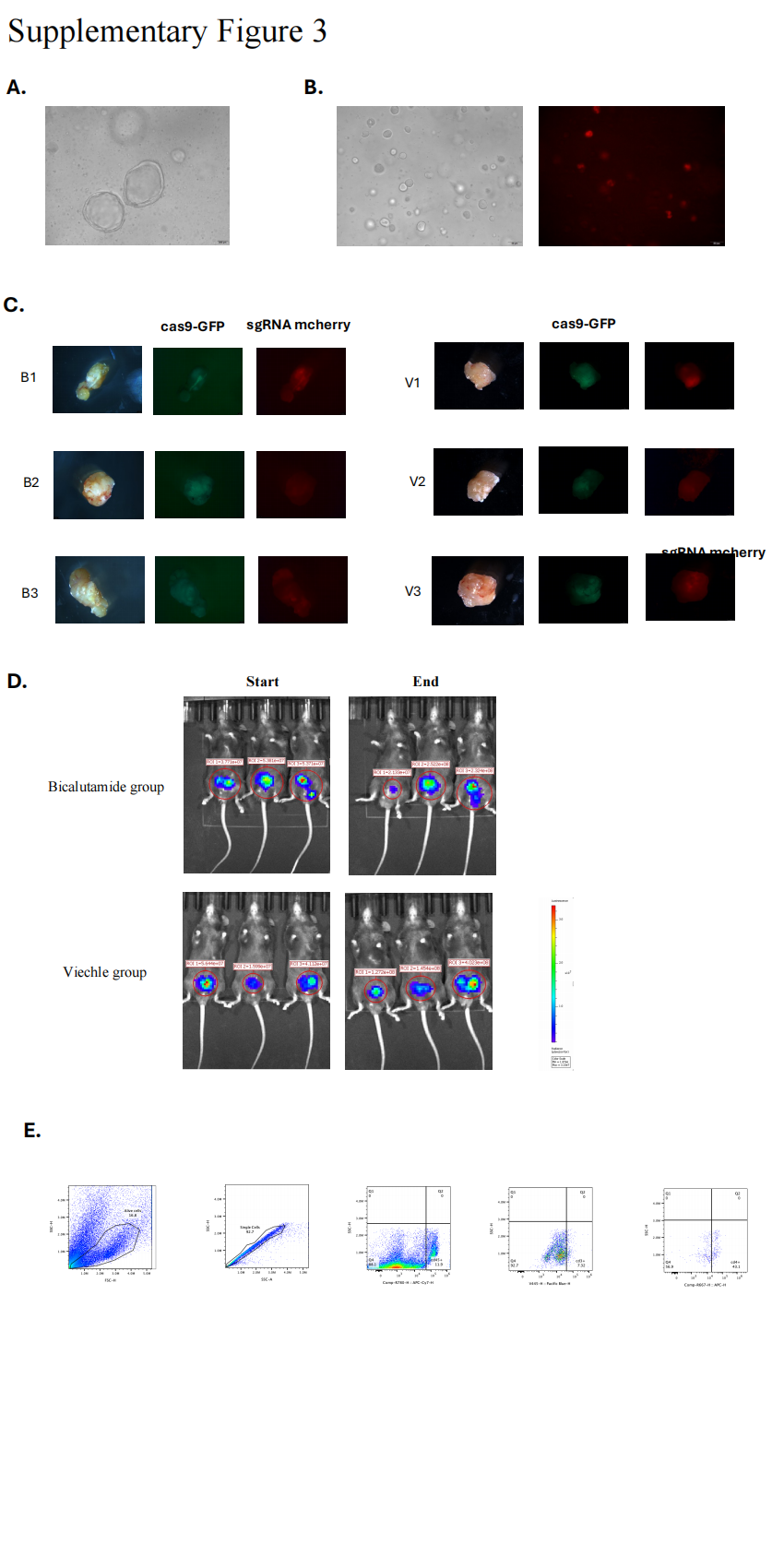

Supplement: Supplementary Figure 3 — (A) Brightfield (BF) images of Normal prostate organoids. (B) BF and fluorescent image of CRISPR-Cas9 gene editing prostate organoids(TP53 null,sgPten,sgRb1,c-myc). (C) BF images of the prostate tumors in bicalutamide group and vehicle group alongside corresponding fluorescent images. (D) Bioluminescent images of mice in bicalutamide group and vehicle group at the start and end of treatment, arranged from left to right as B1, B2, B3, and V1, V2,V3. (E) Representative flow cytometry gating strategy. Live cells were first gated, followed by exclusion of doublets to obtain single cells. From this population, CD45+ cells were selected, and within them, CD3+ T cells were identified. CD4+ T cells were then gated from the CD3+ population. [file Image3.tif]

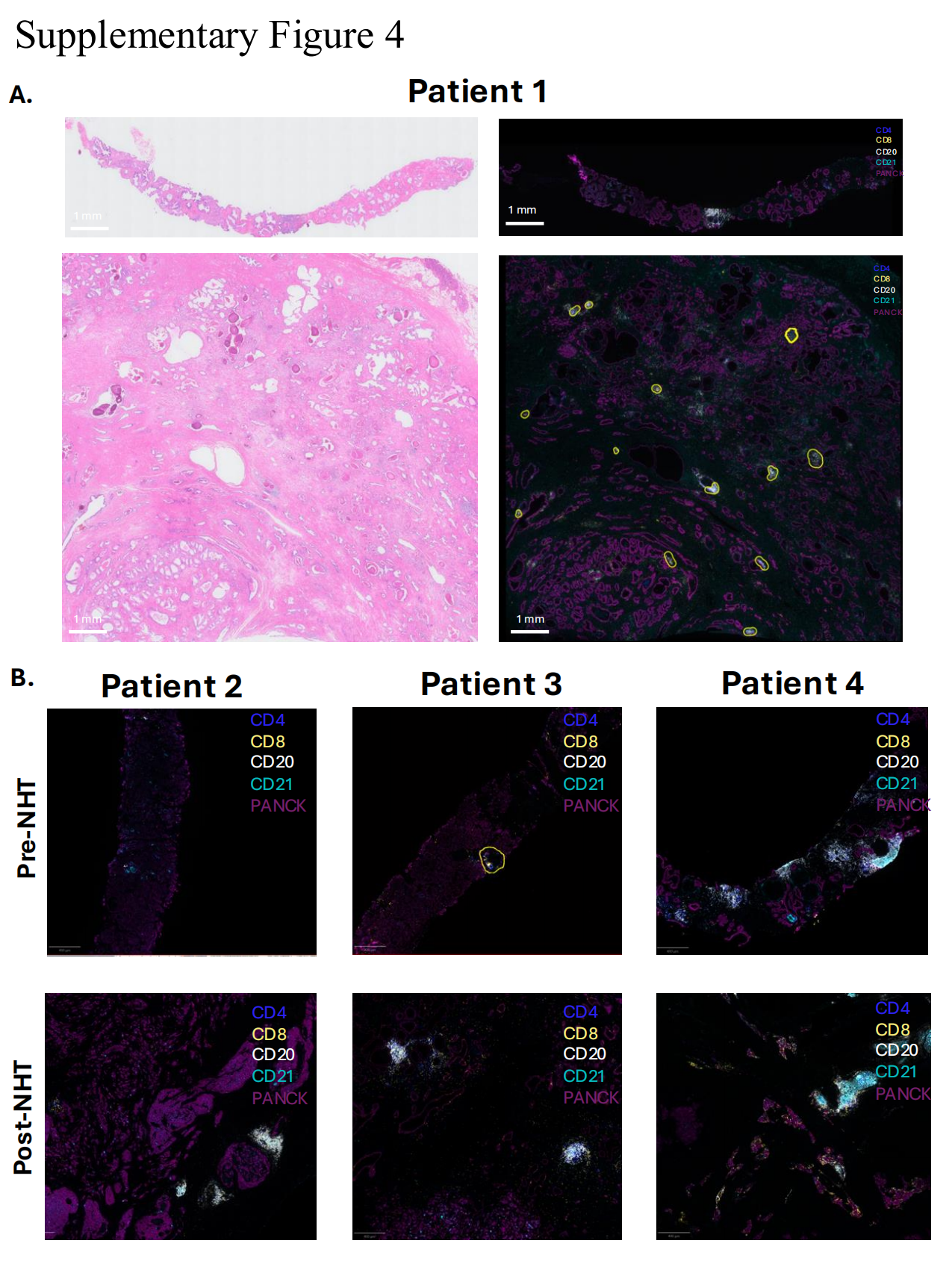

Supplement: Supplementary Figure 4 — Immunohistological Analysis of TLS in cohort 3. A: HE and mIHC staining of paired pre- and post-NHT tumor specimens from Patient 1 in cohort 3; B: Comparison of TLS formation and immune marker distribution in paired pre- and post-NHT samples. [file Image4.tif]

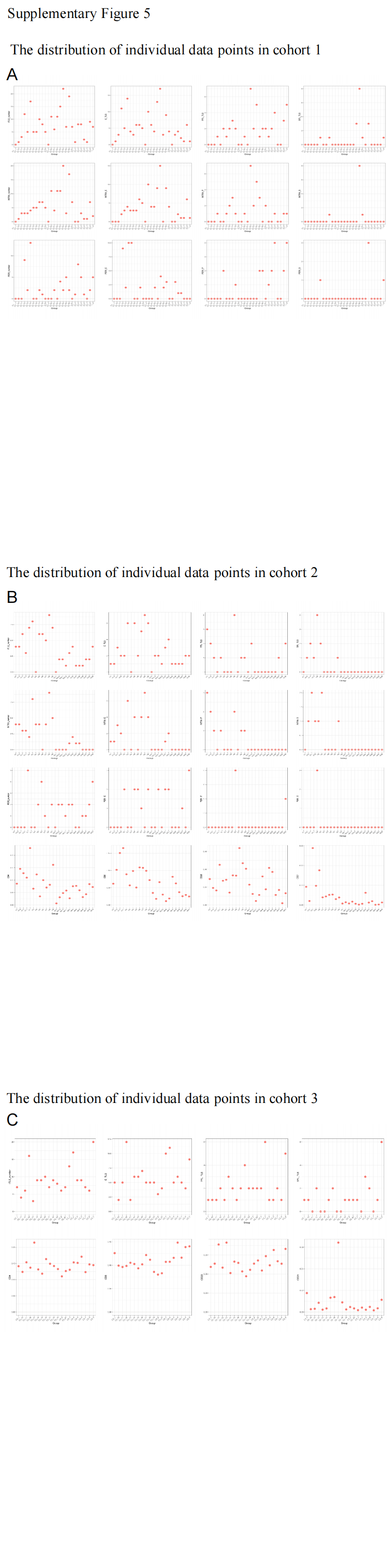

Supplement: Supplementary Figure 5 — The distribution of total TLS characteristics in Cohort 1 (A), Cohort 2 (B) and Cohort 3 (C). tTLS: total tertiary lymphoid structures number, E_TLS: early TLS, PFL_TLS: primary follicle-like TLS, SFL_TLS: secondary follicle-like TLS, INTRA_number: intra-tumoral TLS number, INTRA_E: intra-tumoral early TLS number, INTRA_P: intra-tumoral primaryTLS number, INTRA_S:: intra-tumoral secondary TLS number. PERI_number: peri-tumoral TLS number, PERI_E: peri-tumoral early TLS number, PERI_P: peri-tumoral primaryTLS number, PERI_S: peri-tumoral secondary TLS number. CD4: Ratio of CD4+ cells in tumor micro-environment. CD8: Ratio of CD8+ cells in tumor micro-environment. CD20: Ratio of CD20+ cells in tumor micro-environment. CD21: Ratio of CD21+ cells in tumor micro-environment. [file Image5.tif]

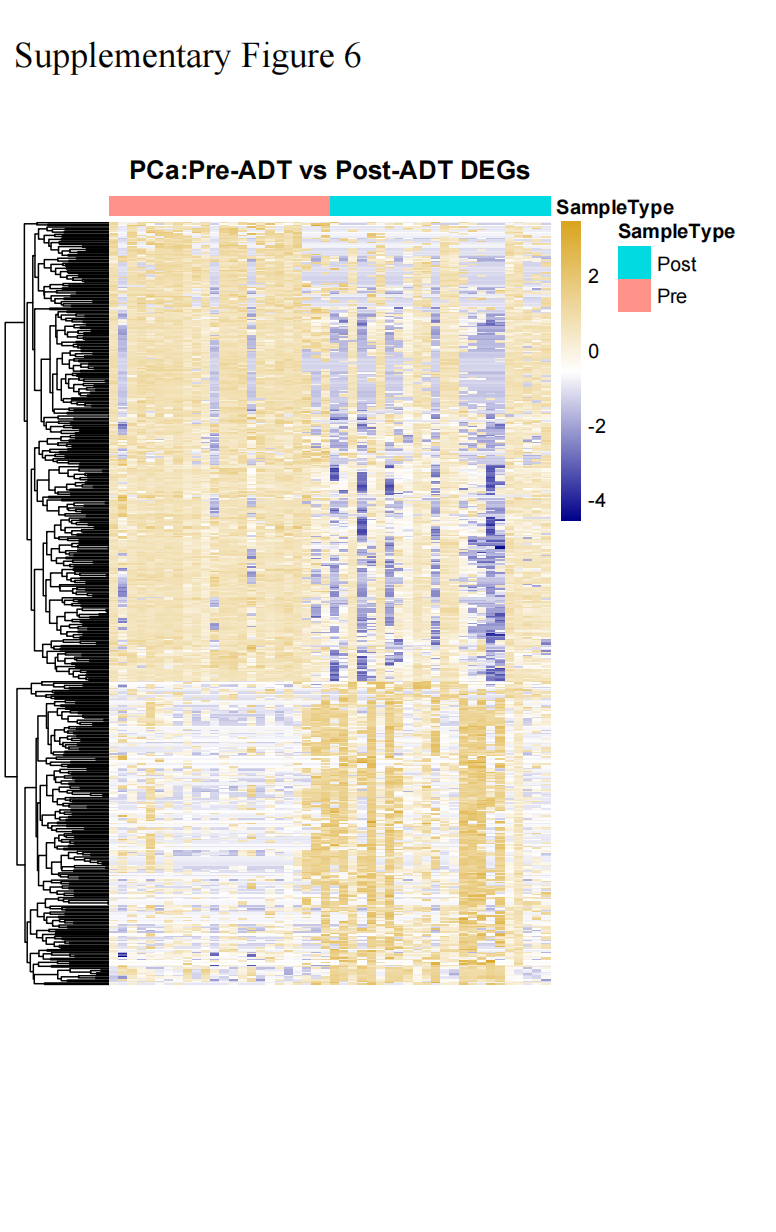

Supplement: Supplementary Figure 6 — The heatmap of differential gene expression analysis pre- and post- NHT treatment via GSE111177 ( Supplementary Table 4 ). [file Image6.tif]
