# Supplementary material for: Characteristics of tertiary lymphoid structures in prostate cancer and the impact of neoadjuvant therapy on their formation and maturation
Source: Front Immunol. 2025 Nov 4;16:1663396. doi: 10.3389/fimmu.2025.1663396 (PMC12623385; doi:10.3389/fimmu.2025.1663396)
Supplement: Supplementary file 8 [file Table1.docx]

**Supplementary Table 1 Baseline clinicopathological characteristics in cohort 1 patients**

|  | **Mature TLS Positive**  **(N=18)** | **Mature TLS Negative**  **(N=9)** | **P** |
| --- | --- | --- | --- |
| **Age, year** | 66.0±8.3 | 66.7±5.6 | 0.29 |
| **PSA, ng/ml** | 17.6±10.3 | 10.9±7.7 | 0.07 |
| **Gleason-grade group at biopsy, n (%)** |  |  |  |
| <7 | 3 | 0 | 0.15 |
| 3+4 | 8 | 2 | 0.35 |
| 4+3 | 3 | 2 | 0.55 |
| >7 | 4 | 5 | 0.08 |
| **Pathological T stage, n, (%)** |  |  |  |
| pT2 | 10 | 6 | 0.45 |
| pT3a | 8 | 3 |  |
| **Positive Margins** |  |  |  |
| Absent | 18 | 8 | 0.33 |
| Present | 0 | 1 |  |
