# Supplementary material for: Characteristics of tertiary lymphoid structures in prostate cancer and the impact of neoadjuvant therapy on their formation and maturation
Source: Front Immunol. 2025 Nov 4;16:1663396. doi: 10.3389/fimmu.2025.1663396 (PMC12623385; doi:10.3389/fimmu.2025.1663396)
Supplement: Supplementary file 9 [file Table2.docx]

Supplementary Table 2: Antibodies list

| **Antibody** | **Source** | **Cat** | **application** |
| --- | --- | --- | --- |
| Anti-CD4 antibody | Abcam | Ab67480 | mIHC |
| CD8a Monoclonal antibody | proteintech | 66868-1-Ig | mIHC |
| CD20 Monoclonal antibody | proteintech | 60271-1-Ig | mIHC |
| CD21 Polyclonal antibody | proteintech | 24374-1-AP | mIHC |
| Ki67 Recombinant Rabbit Monoclonal Antibody | huabio | HA721115 | mIHC |
| pan Cytokeratin Recombinant Mouse Monoclonal Antibody | huabio | HA601094 | mIHC |
| Anti-androgen receptor antibody | abcam | Ab133273 | IHC |
| Anti-prostate specific androgen antibody | abcam | ab53774 | IHC |
| APC anti-mouse CD4 Antibody | BioLegend | 100412 | Flow cytometry |
| APC-Cy7 Rat Anti-Mouse CD45 | BD Pharmingen | 557659 | Flow cytometry |
| Pacific Blue™ anti-mouse CD3 Antibody | BioLegend | 100214 | Flow cytometry |
|  |  |  |  |
