# Supplementary material for: Characteristics of tertiary lymphoid structures in prostate cancer and the impact of neoadjuvant therapy on their formation and maturation
Source: Front Immunol. 2025 Nov 4;16:1663396. doi: 10.3389/fimmu.2025.1663396 (PMC12623385; doi:10.3389/fimmu.2025.1663396)
Supplement: Supplementary file 10 [file Table3.docx]

**Supplementary Table 3: sgRNA sequences**

| sgRNA | Sequence |
| --- | --- |
| sgRb1-F | CACCGACGTTCAGAATCCACGGGA |
| sgRb1-R | AAACTCCCGTGGATTCTGAACGTC |
| sgPten-F | CACCGAACAAAAGGAGATATCAAG |
| sgPten-R | AAACCTTGATATCTCCTTTTGTTC |
